# Lymphome à cellules de la zone marginale splénique

**DOI:** 10.11604/pamj.2017.26.111.11446

**Published:** 2017-03-01

**Authors:** Sinaa Mohamed

**Affiliations:** 1 Service d’Anatomie Pathologique, Hôpital Militaire Moulay Ismail, Meknès, Maroc

**Keywords:** Lymphome de la zone marginale splénique, indolent, pronostic, Splenic marginal zone lymphoma, indolent, prognosis

## Abstract

Le lymphome à cellules de la zone marginale splénique (LZMS) est un lymphome B exceptionnel, bien définie dans la classification OMS 2016 des tumeurs hématopoïétiques, qui peut être source de pièges diagnostiques. Patiente âgée de 72 ans, hospitalisait pour l’exploration d’une énorme splénomégalie confirmée par une tomodensitométrie (TDM). Le bilan biologique était normal. Une splénectomie a été réalisée. L’étude microscopique de la pièce opératoire montrait une prolifération lymphomateuse diffuse à petites cellules, avec un marquage par le CD20. Le CD 5 et le CD 43 sont négatifs. Le diagnostic d’un LZMS a été retenu. Le LZMS est représente moins de 2% de l’ensemble lymphomes non hodgkiniens. Il touche le sujet âgé de plus de 50 ans, caractérisé habituellement par la présence d'une splénomégalie volumineuse sans adénopathies. L'hémogramme montre dans trois quarts des cas la présence inconstante de lymphocytes villeux. Le diagnostic est essentiellement anatomopathologique, il montre une atteinte constante nodulaire ou parfois diffuse de la pulpe blanche du parenchyme splénique. Les cellules tumorales sont de petite taille exprimant les marqueurs lymphoïdes B: CD19, CD20, CD22, CD79. Elles sont négatives pour le CD5, CD10, la cyclinde D1 et le CD43. Aucune anomalie cytogénétique spécifique du LZMS n'a été identifiée. C’est un lymphome indolent dont le traitement est jusqu’à ce jour non codifié, dépend des facteurs pronostiques. Le décès est lié au risque de transformation à un lymphome à grandes cellules.

## Abstract (en)

Splenic marginal zone lymphoma (SMZL) is a rare B-cell lymphoma, well defined according to the 2016 WHO classification of tumors of the hematopoietic tissue, which can raise diagnostic problems. We report the case of a 72-year old patient, hospitalised for exploration of a massive splenomegaly confirmed by computed tomography (CT). Laboratory tests were normal. The patient underwent splenectomy. Microscopic examination of the surgical specimen showed widespread lymphomatous proliferation in small cells associated with CD20 marker. CD 5 and CD 43 were negative. The diagnosis of SMZL was retained. SMZL accounts for less than 2% of non-Hodgkin lymphomas. It affects subjects older than 50 years, and it is usually characterized by the presence of a massive splenomegaly without lymphadenopathies. Blood count shows inconsistent presence of villous lymphocytes in three quarters of cases. The diagnosis is essentially based on anatomopathological evaluation and shows constant nodular involvement or sometimes diffuse involvement of white pulp of the splenic parenchyma.Tumor cells are small in size with B lymphoid markers expression: CD19, CD20, CD22, CD79. They are negative for CD5, CD10, cyclin D1 and CD43. No specific cytogenetic abnormality in SMZL has been identified. SMZL is an indolent lymphoma whose treatment is not codified, depending on prognostic factors. Mortality is related to the risk of its transformation to large cell lymphoma.

## Introduction

Le lymphome à cellules de la zone marginale splénique (LZMS) est un lymphome non hodgkinien (LNH) B à petites cellules de bas grade très rare, qui représente 1 à 2 % de l’ensemble des LNH [1]. Il s’agit d’une entité bien distincte dans la classification de l’OMS 2016 des tumeurs hématopoïétiques, qui pose parfois un problème de diagnostic différentiel avec d’autres lymphomes indolents [1]. Il est caractérisé par une splénomégalie isolée sans adénopathies, parfois associé à une lymphocytose sanguine modérée.

## Patient et observation

**Figure 1:**
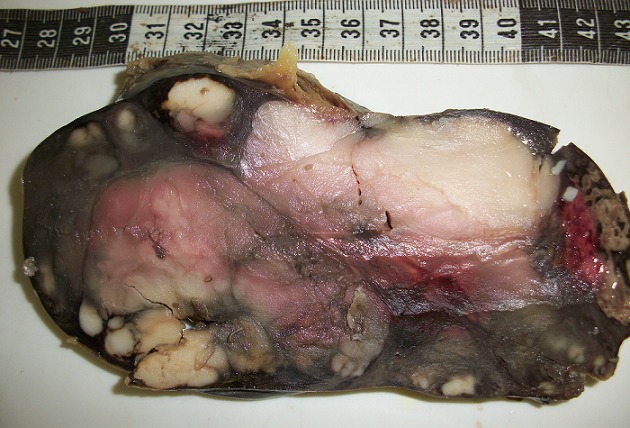
Aspect macroscopique multinodulaire de la rate

**Figure 2:**
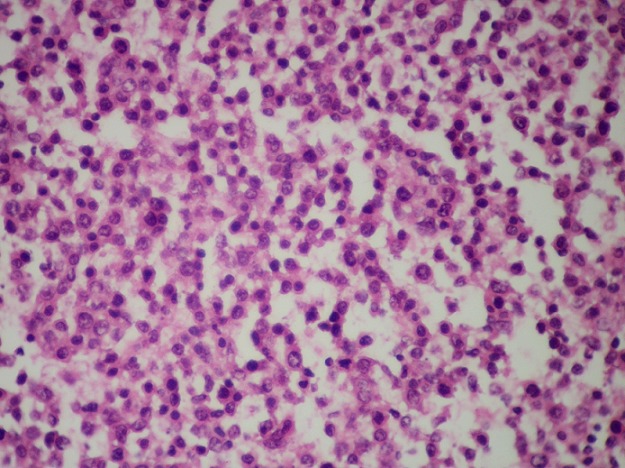
Prolifération lymphomateuse à petites cellules B au cytoplasme réduit amphophile et aux noyaux assez homogène: LZMS (x200)

**Figure 3:**
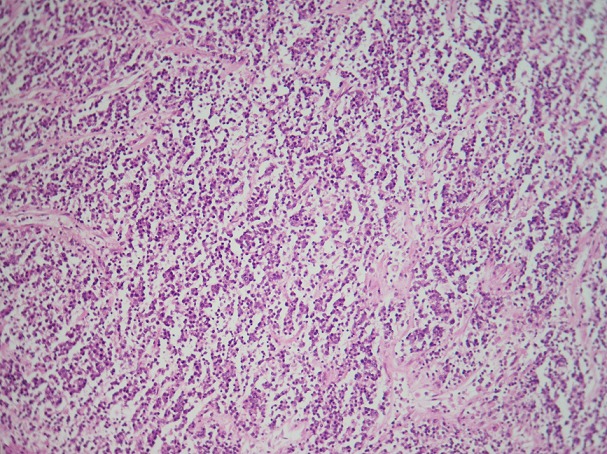
Infiltration sinusoïdale de la pulpe rouge de la rate (x100)

**Figure 4:**
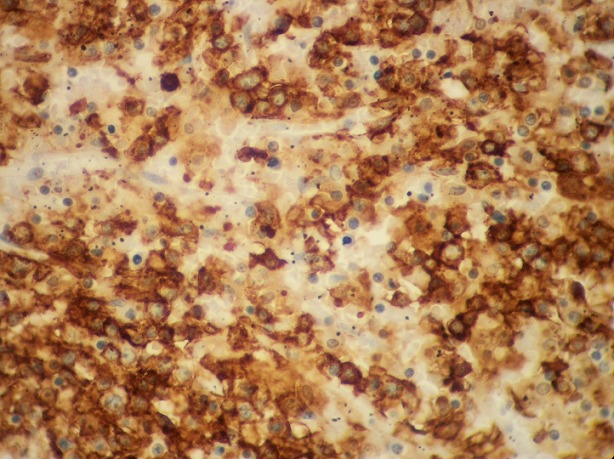
Marquage positif par l’anticorps anti CD20 (x200)

**Figure 5:**
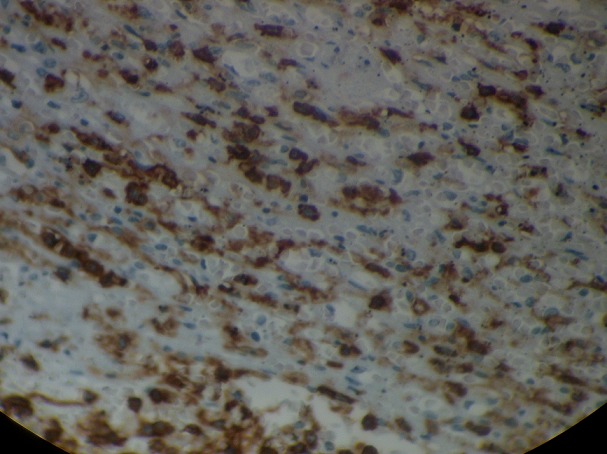
Marquage positif des plasmocytes par l’anticorps anti DBA44 (x200)

**Figure 6:**
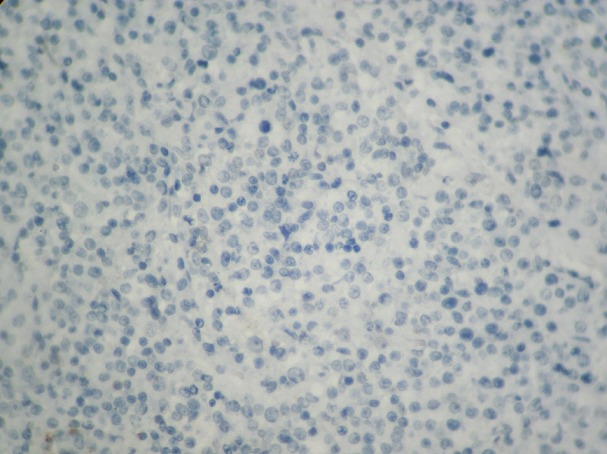
Absence de marquage par l’anticorps anti CD5 (x200)

**Figure 7:**
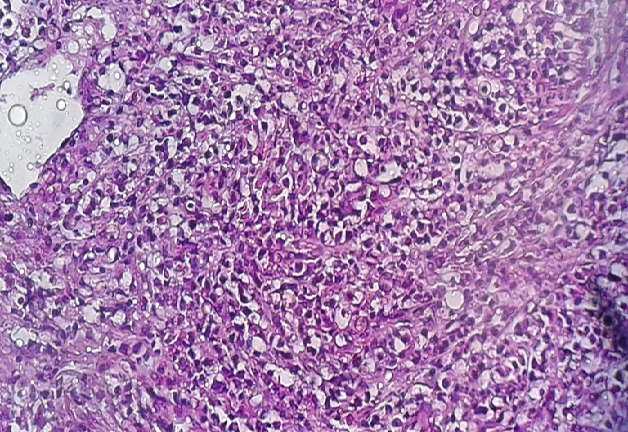
Envahissement médullaire pas amas de lymphocytes B de petite taille (x200)

Il s’agit une patiente âgée de 72 ans, hospitalisée pour douleur de l’hypochondre gauche évoluant depuis 3ans, avec vomissements postprandiaux évoluant dans un contexte d’amaigrissement non chiffré. L’examen clinique trouve une splénomégalie. Les aires ganglionnaires sont libres. L’hémogramme ne trouve pas de lymphocytes villeux. Le scanner trouve une énorme splénomégalie nodulaire d’aspect tumoral. Une splénectomie à visée diagnostique et thérapeutique a été réalisée. Notre laboratoire a reçu une pièce de splénomégalie pesant 1820g et mesurant 25x15x7,5 cm avec à la coupe présence d’un processus tumoral d’aspect multi nodulaire, de couleur blanc grisâtre (Figure 1). L’examen microscopique montre une prolifération lymphomateuse diffuse parfois nodulaire, faite de cellules de taille petite, au cytoplasme réduit amphophile et aux noyaux irréguliers (Figure 2), avec la présence en périphérie d’une infiltration sinusoïdale (Figure 3). Le complément immunohistochimique a montré une positivité des cellules tumorales pour le CD20 (Figure 4) et le DBA44 (Figure 5), elles sont négatives pour le CD5 (Figure 6), CD10, CD23 et le CD43. Le diagnostic d’un lymphome splénique de la zone marginale a été retenu. Un bilan d’extension a été réalisé notamment une BOM, il a montré un envahissement médullaire (Figure 7).

## Discussion

Décrit pour la première fois en 1992 [1], le LZMS est un lymphome B non hodgkinien indolent primitivement splénique [1] qui représente 1 à 2 % de l’ensemble des lymphomes non hodgkiniens [1]. Il touche préférentiellement les sujets de plus de 50 ans avec un âge moyen au diagnostic de 65 ans et un sex-ratio de 1. Dans certaines séries, les LZMS semblent fréquemment associés à une infection chronique par le VHC. D’autres auteurs ont montré quel est associé à une infection chronique active par le VHC régressent sous traitement antiviral [1, 2]. La présentation habituelle est celle d’un syndrome tumoral limité avec une splénomégalie isolée, parfois associée à des adénopathies du hile splénique [1]. Plus rarement sont rapportées une hépatomégalie et des adénopathies intraabdominales (10% des cas). L’infiltration médullaire est quasi constante, 95% des LZMS étant d’emblée de stade IV au diagnostic [2]. L’hémogramme retrouve des cellules lymphomateuses circulantes dans 64% des cas, prenant parfois l’aspect de lymphocytes villeux [1–3]. Une ou plusieurs cytopénies sont fréquemment retrouvées, en rapport avec un hypersplénisme, un mécanisme auto-immun ou, un mécanisme central par envahissement médullaire. L’immunoélectrophorèse des protéines révèle une protéine monoclonale dans un tiers des cas, de type IgM le plus souvent, et dépassant rarement 30g/l [2]. Le frottis sanguin montre la présence inconstante de lymphocytes villeux.

Le critère diagnostique le plus fiable est l’obtention d’une histologie splénique typique, ou en l’absence de splénectomie, la présence d’un clone lymphocytaire B circulant et présentant une morphologie villeuse [1, 3]. Les cellules tumorales expriment un des marqueurs lymphoïdes B : CD19, CD20, CD22, et CD79b. Elles sont négatives pour le CD5, CD10, Cycline D1 et le CD43. S´il existe des cas indiscutables de LZMS CD5+, le diagnostic doit être néanmoins retenu avec une extrême prudence et il convient d´éliminer dans ces cas inhabituels les diagnostics de leucémie lymphoïde chronique ou de phase pré-leucémique de lymphome à cellules du manteau. L´expression du DBA44 est observée dans environ 80 % des cas [1, 4]. Une expression du CD11c est souvent présente, mais de façon inconstante (47 %) [1, 4]. L’atteinte splénique exclusive reste rare mais non exceptionnelle, comme le montre notre observation. Dans certains cas, l’immunophénotypage sur sang et la BOM sont tous les deux négatifs, et seul l’examen anatomopathologique de la pièce de splénectomie permet de poser le diagnostic [1, 5]. Celui-ci montre une prolifération touchant la zone marginale périfolliculaire qui détruit le manteau, entourant et remplaçant les centres germinatifs de la pulpe blanche résiduelle. L’infiltration sinusoïdale est très caractéristiques. Ce sont de petites cellules rondes au cytoplasme assez abondant, et aux noyaux irréguliers, parfois villeux, qui colonisent les follicules, entourées par des cellules plus grandes à cytoplasme clair qui ressemblent aux cellules de la zone marginale [1, 6]. Les LZMS s’associent dans 20% des cas à des manifestations auto-immunes [1, 2, 4]. Cette auto-immunité est souvent de traduction biologique isolée et multiple chez un même patient: thyroïdite auto-immune et anticorps antinucléaires, pancytopénie auto-immune, facteur rhumatoïde et anticorps antinucléaires, syndrome des antiphospholipides, maladie de Still de l’adulte, anticorps anticoagulant circulant de type lupique et syndrome de Gougerot Sjögren (SGS) [1, 2, 4, 7] Aucune anomalie cytogénétique spécifique du LSZM n´a été identifiée, néanmoins Les délétions en 7q ainsi que Les translocations impliquant les gènes des chaines Iourdes des immunoglobulines dont les t(11 ;14) (q3;q32), sont les anomalies les plus fréquentes [1, 8].

L’évolution est indolente avec une médiane de survie de 8 à 13 ans selon les séries, et une survie à cinq ans de 76% [1, 9]. Le décès est lié à la progression du lymphome ou à sa transformation en lymphome diffus à grandes cellules qui survient dans environ 10% des cas [10, 11]. Les facteurs pronostiques identifiés ne sont pas les mêmes selon les séries. Thieblemont et al. [7] montrent que la survenue de manifestations auto-immunes et l’existence d’une protéine monoclonale sont associées à un délai de progression du lymphome plus court. Dans la série d’Arcaini et al. [12] portant sur un plus grand nombre de patients (309 cas), les facteurs péjoratifs identifiés sont l’existence d’une anémie (hémoglobine inférieure à12g/dl), d’un taux de LDH augmenté, et d’un taux d’albumine diminué (<3,5g/dl) [1, 12].

À ce jour, Le traitement reste encore assez mal codifié, il varie entre l’abstention thérapeutique, la splénectomie, la chimiothérapie (anthracyclines, fludarabine) et/ou l’irradiation splénique. Aucun traitement n’a permis d’allonger significativement la survie des patients atteints de LZMS. Si les LZMS associés au virus de l’hépatite C relèvent en premier lieu d’un traitement antiviral, la splénectomie reste le traitement de choix en cas de cytopénie ou de splénomégalie importante, la chimiothérapie étant proposée en cas de contre-indication à la chirurgie ou de progression clinique après splénectomie. La chimiothérapie adjuvante après splénectomie en cas de facteurs de mauvais pronostic (signes généraux d’évolutivité, adénopathies abdominales, augmentation des LDH, présence de grandes cellules à l’histologie splénique) augmente le taux de rémission complète mais ne modifie pas la survie, ni le risque de transformation et de rechutes [1].

## Conclusion

Le lymphome splénique de la zone marginale est un lymphome indolent, dont le traitement est jusqu’à présent non standardisé, dépend essentiellement des facteurs pronostiques. La survie globale est de 76% à 5 ans. Le décès est lié au risque de transformation à une hémopathie à grandes cellules.
